# An influenza-derived membrane tension-modulating peptide regulates cell movement and morphology via actin remodeling

**DOI:** 10.1038/s42003-019-0486-3

**Published:** 2019-06-26

**Authors:** Toshihiro Masuda, Kentarou Baba, Takeshi Nomura, Kazuya Tsujita, Tomo Murayama, Toshiki Itoh, Tomoka Takatani-Nakase, Masahiro Sokabe, Naoyuki Inagaki, Shiroh Futaki

**Affiliations:** 1 Institute for Chemical Research, Kyoto University, Uji, Kyoto 611-0011 Japan; 2 Division of Biological Science, Nara Institute of Science and Technology, Ikoma, Nara 630-0192 Japan; 3 Department of Agro-environmental Sciences, Kyushu University, Fukuoka, 819-0395 Japan; 4 Division of Membrane Biology, Biosignal Research Center, Kobe University, 1-1 Rokkodai-cho, Nada-ku, Kobe, Hyogo 657-8501 Japan; 5 Department of Biochemistry and Molecular Biology, Kobe University Graduate School of Medicine, 7-5-1 Kusunoki-cho, Chuo-ku, Kobe, Hyogo 650-0017 Japan; 6 Mechanobiology Laboratory, Nagoya University Graduate School of Medicine, 65 Tsurumai, Nagoya, 466-8550 Japan; 7 School of Pharmacy and Pharmaceutical Sciences, Mukogawa Women’s University, Nishinomiya, Hyogo 663-8179 Japan

**Keywords:** Lamellipodia, Membranes

## Abstract

Tension in cell membranes is closely related to various cellular events, including cell movement and morphogenesis. Therefore, modulation of membrane tension can be a new approach for manipulating cellular events. Here, we show that an amphipathic peptide derived from the influenza M2 protein (M2[45–62]) yields lamellipodia at multiple sites in the cell. Effect of M2[45–62] on cell membrane tension was evaluated by optical tweezer. The membrane tension sensor protein FBP17 was involved in M2[45–62]-driven lamellipodium formation. Lysine-to-arginine substitution in M2[45–62] further enhanced its activity of lamellipodium formation. M2[45–62] had an ability to reduce cell motility, evaluated by scratch wound migration and transwell migration assays. An increase in neurite outgrowth was also observed after treatment with M2[45–62]. The above results suggest the potential of M2[45–62] to modulate cell movement and morphology by modulating cell membrane tension.

## Abstract (web summary)

Toshihiro Masuda et al. find an amphiphilic peptide derived from the influenza M2 protein, M2[45–62], which causes small actin-rich lamellipodia at multiple sites in the cell, inhibiting cell movement by decreasing membrane tension. They show that M2[45–62] stimulates actin polymerization in rat hippocampal neurons.

## Introduction

Tension in cell membrane (plasma membrane) is involved in regulation of various vital biological processes^1^. Cell movement is an example that is affected by cell membrane tension. Defects in cell movement are linked to various pathological processes, including neurodevelopmental disorders and tumor progression and metastasis^2^. Cell membrane tension is also intimately linked to cell morphogenesis. Development of new molecular tools that regulate cell membrane tension should have a great potential to understand the fundamental aspects of cell homeostasis and associated disorders. Such cell manipulation tools may also hold promises for cell engineering and therapeutic applications.

**Fig. 1:**
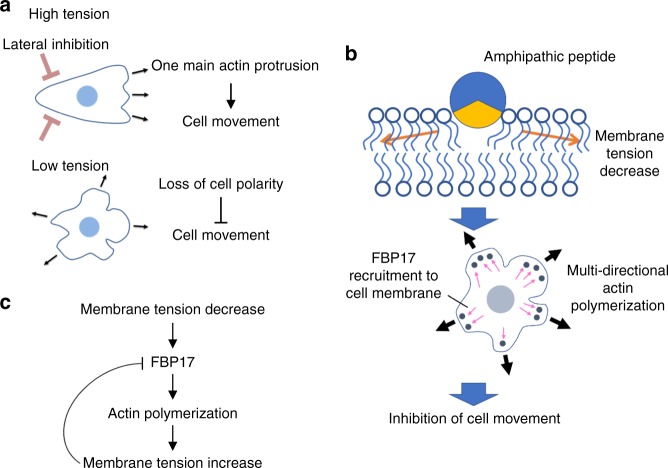
**a** Cell membrane tension as a master regulator of cell shape. Lateral protrusions were suppressed at high tension in favor of one main protrusion, pointing in the direction of migration, therefore increasing the efficiency of movement. A reduction in cell membrane tension allowed lamellipodia to form in multiple directions, reducing the polarity of the cell and its movement. **b** Working hypothesis of cell movement inhibition by amphipathic peptides. The amphipathic peptide is inserted into the membrane to expand the surface area of the cell membrane, which decreases cell membrane tension. The decrease in cell membrane tension leads to lamellipodium formation in multiple directions, resulting in inhibition of cell movement via FBP17. **c** Feedback regulation of cell membrane tension by FBP17. FBP17 is recruited to the cell membrane after sensing a reduction in cell membrane tension, thereby promoting actin polymerization. This leads to an increase in cell membrane tension, which releases FBP17 from the cell membrane and suppresses FBP17 from stimulating actin polymerization. Thus, FBP17 may play an important role in sensing and translating cell membrane tension into cell movement and defining front-rear polarity in moving cells

Cell membrane tension is generated from the in-plane tension in the lipid bilayer and from the adhesion between the membrane and the cytoskeleton, and is also referred as effective membrane tension^1^. It has been suggested that the interplay between cell membrane tension and the organization of cytoskeletal protein regulates cell movement and morphogenesis^3^. Cell membranes are considered obstacles to polymerization of cytoskeletal protein actin^3–6^. It was demonstrated that the tension in cell membranes resisted membrane extension and was a critical factor controlling cell movement and morphogenesis by maintaining cell polarity^7–10^. An increase in cell membrane tension enhances motility by suppressing lateral actin polymerization and by stabilizing the main protrusion toward the direction of migration (Fig. 1a). In contrast, a decrease in cell membrane tension yields small actin-rich veils called lamellipodia at multiple sites in the cell, decreasing cell polarity, and cell displacement speed (Fig. 1a). Therefore, the molecules that regulate cell membrane tension may be screened in terms of their effect on actin organization.

Although the importance of cell membrane tension in regulating cell movement and morphology has been reported^1^, only approaches to use small-molecular-weight amphiphiles, including deoxycholic acid, have been reported to reduce cell membrane tension^11,12^, but no approach by externally added peptides or proteins as long as we know. Such peptides should have a great promise as a novel chemical tool to modulate cell function, with potential of conjugating other cellular or non-cellular functional proteins. Considering the case of deoxycholic acid, we hypothesized that amphipathic peptides derived from membrane remodeling proteins may also have an ability to decrease cell membrane tension by inserting the amphipathic segments into the lipid bilayer (Fig. 1b)^13^.

Here, we demonstrate that cell membrane tension was successfully modulated by a synthetic 18-residue peptide derived from the influenza M2 protein (amino acid positions 45–62) (M2[45–62]). This peptide was obtained through the evaluation of the effect on actin organization. M2[45–62] decreased cell polarity by inducing lamellipodium formation from the cell membrane in multiple directions, thus inhibiting cell movement. An involvement of the F-BAR domain protein FBP17 in this process was confirmed by siRNA knockdown—FBP17 has been known as a sensor of membrane curvature and was recently reported to also have a role as a membrane tension sensor, translating cell membrane tension into cell movement (Fig. 1b, c)^10^. A reduction in cell membrane tension was confirmed by an optical tweezer analysis. M2[45–62] also affected threshold pressure to open the bacterial mechanosensitive channels MscS and MscL. There was a significant suppression of cell motility by M2[45–62], which was evaluated by scratch wound migration and transwell migration assays. Morphological changes, including increased neurite outgrowth, in primary neuronal cultures were induced by treatment with M2[45–62]. These results suggest promise for our peptide-mediated approach to modulate cell movement and morphogenesis by affecting cell membrane tension.

## Results

### Amphipathic peptides derived from membrane remodeling proteins

To establish the validity of our approach, amphipathic segments from seven membrane remodeling proteins (Bin1, M2, Epsin, MIM, Arf, dAmph, and SH3YL1) were selected as candidate peptides to alter cell membrane tension^14–20^ (Table 1). These proteins, from which the respective amphipathic segments were derived, are involved in membrane remodeling processes, including endocytosis, budding and vesicle trafficking. Each amphipathic segment has an important role in the interaction between the respective protein and the membrane. These peptides were prepared using Fmoc-solid-phase peptide synthesis^21^. As methionine is subject to oxidation, the methionine residues were replaced by norleucine (X) for ease of handling.

### Effects of amphipathic peptides on F-actin distribution

If these amphipathic peptides have the ability to influence cell membrane tension, treating the cells with the peptides should affect the mode of actin polymerization. COS-1 cells were treated with the amphipathic peptides dissolved in isotonic buffer for 15 min. The cells were fixed, and the F-actin structures in the cells were monitored by confocal laser scanning microscopy (CLSM) using rhodamine-labeled phalloidin, an F-actin binding peptide^22^. Prior to the experiments, cytotoxicity induced by the peptides was evaluated using the WST-1 cell proliferation assay^23^ (Supplementary Fig. 1) to ensure cell viability, and those peptide concentrations that preserved cell viability above 90% (i.e., 10 µm for M2[45–62], 20 µm for Arf[1–17] and dAmph[3–28], and 40 µm for the other peptides) were employed for subsequent studies.

**Fig. 2:**
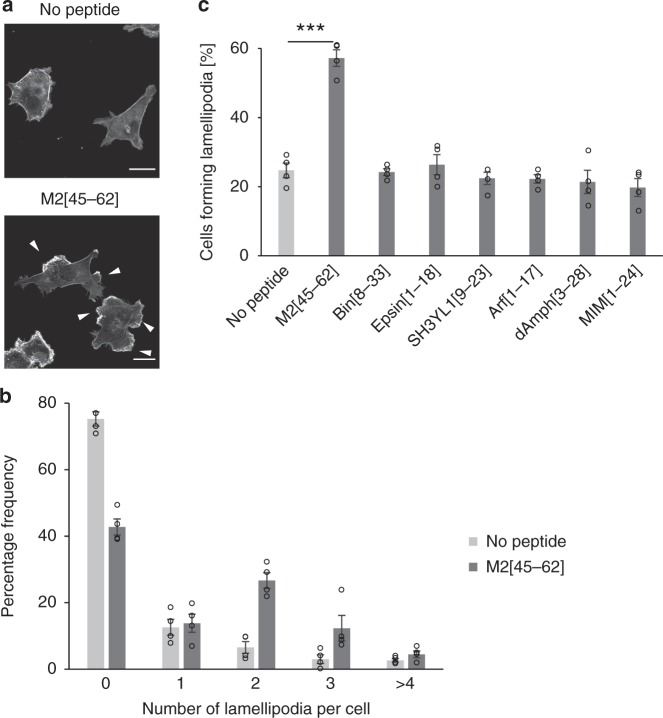
M2[45–62] induced lamellipodium formation in COS-1 cells. **a** Confocal laser scanning microscopy images of F-actin (stained with rhodamine–phalloidin) in COS-1 cells treated with M2[45–62] (10 µm) for 15 min. Arrowheads indicate lamellipodia. Lamellipodium formation in multiple directions was predominantly observed in M2[45–62]-treated cells. Scale bar, 20 µm. **b** Distribution of the number of lamellipodia per cell after treatment with M2[45–62]. No peptide denotes cells treated with buffer without M2[45–62]. The means ± standard error were derived from ~ 200 cells pooled from four independent experiments. The number of cell populations bearing more than one lamellipodium per cell increased in response to the M2[45–62] treatment. **c** Quantification of lamellipodium formation after amphipathic peptide treatment (M2[45–62]: 10 µm; Arf[1–17] and dAmph[3–28]: 20 µm; other peptides: 40 µm). The means ± standard error were derived from ~ 200 cells pooled from four independent experiments. ***P < 0.001, Student’s *t* test

There was a significant increase in the population of cells with lamellipodia following treatment with M2[45–62] (Fig. 2a, b), whereas no marked lamellipodium formation was observed in cells treated with other peptides or in the control cells (i.e., cells treated with buffer without peptides) (Fig. 2a). Figure 2c shows the percentage of cells that formed lamellipodia (see Supplementary Fig. 2 for representative images of cells bearing each number of lamellipodia). Whereas lamellipodium formation was observed in ~ 60% of M2[45–62]-treated cells, lamellipodia were observed in only ~ 25% of the control cells (no peptide) or cells treated with other peptides. These results indicated the ability of M2[45–62] to induce actin reorganization.

The finding that M2[45–62] had more interactions with the membrane compared with the other peptides was supported by an analysis of the interaction between nitrobenzoxadiazole (NBD)-labeled peptides and liposomes. NBD fluorescence reflects the environment in which the NBD group is located, displaying higher quantum yield and a blue shift in the maximal emission wavelength in more hydrophobic environments. Therefore, inserting NBD-labeled peptides into the lipid bilayer should increase fluorescence intensity. Liposomes comprised of 1-palmitoyl-2-oleoylphosphatidylcholine, 1-palmitoyl-2-oleoylphosphatidylglycerol, and cholesterol at a mixing molar ratio of 4:1:2 and varying concentrations were added to the NBD-labeled peptides (0.5 µm). The increase in fluorescence intensity was plotted as a function of the lipid/peptide molar ratio. NBD-labeled M2[45–62] yielded the most notable increase in fluorescence (Supplementary Fig. 3a).

The cell surface interaction of M2[45–62] was also analyzed by CLSM analysis. COS-1 cells treated with NBD-labeled M2[45–62] exhibited marked fluorescent signals at the periphery of the cells, suggesting cell membranes. It has been reported that adding dithionite chemically quenches the NBD groups in the outer leaflets of the bilayers^24^. An immediate decrease in NBD fluorescence was also observed after adding dithionite, suggesting the cell surface localization of NBD-labeled M2[45–62] (Supplementary Fig. 3b). On the other hand, No significant fluorescent signals were observed on the cell membranes after adding NBD-labeled Arf[1–17] (Supplementary Fig. 3c), although the ability of this peptide to bind to liposomes followed that of M2[45–62] (Supplementary Fig. 3a).

Although the extracellular leaflet of cell membrane has been considered predominantly comprised of zwitter-ionic, neutral lipids such as phosphatidylcholine, the extracellular leaflet still contains a few percent of anionic lipids^25^. M2[45–62] had higher cationic charges than the other peptides evaluated and relatively high hydrophobicity (Supplementary Table 1). The potential amphiphilic helical structure of M2[45–62] should also be preferable for the hydrophobic interaction with membranes^26–28^ (see Supplementary Fig. 4). These physicochemical properties of M2[45–62] may yield more membrane interactions and eventually a higher percentage of cells forming lamellipodia compared with other peptides studied. Other peptides than M2[45–62] used in this study were derived from cytoplasmic, curvature-inducing proteins. Inner leaflet of cell membranes is abundant of negatively charged lipids such as phosphatidylserine and phosphatidylinositols, and anionic lipids may be needed for their interaction with cell membranes. It might be possible that these peptides have an activity to alter actin organization, if these peptides interact from cytoplasmic side of cell membranes.

The possibility that M2[45–62] directly targets the membrane bilayer was supported by a study using the D-amino acid version of M2[45–62] [D-M2: rlffkciyrrfkyGlkrg-amide (lower case letters represent d-amino acids)]. If the lamellipodia are formed by the M2[45–62] treatment via interaction with membrane proteins (e.g., receptors and transporters), the M2[45–62] enantiomer should have less activity. However, D-M2 induced marked lamellipodium formation similar to that induced by M2[45–62] (Supplementary Fig. 5).

Distorting the amphiphilic structure of M2[45–62] decreased the membrane interactions and formation of lamellipodia. Scr-M2, bearing the scrambled sequence of M2[45–62] (FRYGRIFLKYKFCKGRLR-amide; Supplementary Fig. 6), was prepared. Scr-M2 treatment yielded fewer cells bearing lamellipodia than did M2[45–62] treatment (Supplementary Fig. 5), suggesting the importance of the amphiphilic structure of the M2[45–62] sequence for lamellipodium formation.

In addition, the presence of serum did not affect lamellipodium formation by the M2[45–62] peptide (Supplementary Fig. 7); there was no marked difference in lamellipodium formation in cells treated with M2[45–62] in the presence versus absence of serum.

### Membrane tension changes induced by M2[45–62]

**Fig. 3:**
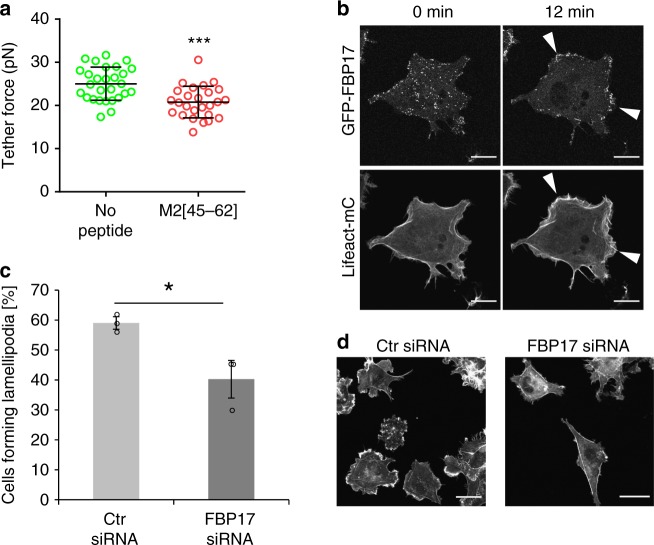
M2[45–62] reduces cell membrane tension, and FBP17 plays an important role in M2[45–62]-driven lamellipodium formation. **a** Measurement of the tether force in COS-1 cells. The tether force of identical cells was measured after treatment with phosphate buffered saline (PBS) or M2[45–62] (20 µm). The mean ± standard deviation were derived from data pooled from three independent experiments. In total, 28 cells (no peptide = PBS treatment) and 26 cells (M2[45–62] treatment) were analyzed. ***P < 0.001, Student’s *t* test. **b** Time-lapse images of COS-1 cells co-expressing GFP–FBP17 and Lifeact-mCherry after addition of M2[45–62] (20 µm). Arrowheads indicate lamellipodia. Scale bar, 20 µm. **c** Quantification of the lamellipodia formed after treatment with control (Ctr) siRNA or FBP17 siRNA. The mean ± standard error were derived from ~ 250 cells pooled from three independent experiments. *P < 0.05, Student’s *t* test. **d** Confocal laser scanning microscopy images of F-actin (stained with rhodamine–phalloidin) in control (Ctr) siRNA or FBP17 siRNA-treated COS-1 cells. Cells were treated with M2[45–62] for 15 min. Scale bar, 30 µm

Lamellipodia forming from cell membranes in multiple directions inhibit cell movement, and a reduction in cell membrane tension is considered to be highly related to this step^7^. We analyzed whether M2[45–62] can reduce cell membrane tension using optical tweezers. Optical tweezers have been employed previously to analyze changes in cell membrane tension^29–31^. Significantly lower tether forces were recorded in M2[45–62]-treated cells than in cells without M2[45–62] treatment (Fig. 3a). This finding suggests that M2[45–62] may have the ability to reduce cell membrane tension via insertion into the membrane. In addition, Scr-M2 and Arf[1–17], which had no apparent lamellipodia formation ability (Fig. 2b and Supplementary Fig. 5), yielded less extent of decrease in tether force to that obtained by M2[45–62], suggesting their inferiority in decreasing cell membrane tension (Supplementary Fig. 8).

The effect of M2[45–62] on membrane tension was further confirmed through the opening behavior of mechanosensitive ion channels, which responds to stretch forces in the membrane lipid bilayer. The mechanosensitive channels of large conductance (MscL) and small conductance (MscS) are well-characterized bacterial mechanosensitive ion channels and are activated by increased membrane tension^32,33^. A patch-clamp system was employed to detect ion flux through a bacterial ion channel in the membrane (Supplementary Fig. 9a). Suction in the pipette leads to an extension of the patch membrane surrounded by the gigaseal. The channels open (or become activated) when extension of the membrane reaches threshold of the channel gating (Supplementary Fig. 9b). Whereas no effect was observed on the threshold pressure (~  −200 mmHg) for MscL activation in the absence of M2[45–62], there was a gradual decrease in the channel activation threshold, reaching a plateau of ~ − 160 mmHg in the presence of M2[45–62] (40 µm) (Supplementary Fig. 9c). A similar tendency was observed for activation of MscS; the activation threshold of ~ − 120 mmHg in the absence of M2[45–62] reached ~ − 100 mmHg in the presence of the peptide (Supplementary Fig. 9c). The above results clearly show the effect of M2[45–62] on membrane tension. However, the decrease in the threshold pressures suggest the increase in membrane tension, which is apparently contradictory to afore mentioned interpretation of the peptide effect on membrane tension. This may be attributed to the experimental setup of mechanosensitive ion channel analysis that immobilizes the outer leaflet of the membrane attached to the pipette. Interaction of M2[45–62] with one side of the clamped membrane leads to an expansion of the leaflet, which in turn stretches the leaflet in the other side of mechanically isolated patch membrane leading to an increase in the tension. In other words, such an asymmetric packing of the lipid bilayer (or peptide interaction with membrane) will yield tension increase in the less packed leaflet (i.e., in the other side of membrane of peptide interaction) as has been reported very recently^34^. On the other hand, in the analysis using optical tweezers, membrane is not immobilized or clamped. Interaction with M2[45–62] should cause an increase in the area of outer leaflet of the cell membrane with less stress in the inner leaflet, probably owing to easy deformation of the inner leaflet or lipid supply from cytoplasm, leading to rather a decrease in cell membrane tension.

Asymmetric insertion of M2[45–62] into cell membranes may yields membrane structural alteration or spontaneous curvature. It has also been reported that spontaneous curvature induction in cell membranes leads to decrease in cell membrane tension^35,36^. Therefore, curvature induction by the membrane interaction with M2[45–62] may be involved in reduction in cell membrane tension induced by M2[45–62].

Hypotonic buffer treatment increases membrane tension^37,38^, which may suppress the lamellipodium formation activity of M2[45–62] by preventing the reduction in M2[45–62]-induced cell membrane tension. Treatment with M2[45–62] in isotonic buffer resulted in the formation of lamellipodia in multiple direction within 15 min (Supplementary Fig. 10a); F-actin organization in live cells was monitored by Lifeact-mCherry (a fusion protein of the F-actin-binding peptide Lifeact to the mCherry fluorescent protein) fluorescence. Alternatively, adding hypotonic buffer abrogated the effect of M2[45–62]. Marked reorganization of F-actin structures was observed, resulting in integration of lamellipodia into one main site (Supplementary Fig. 10b). The inhibition of M2[45–62]-mediated lamellipodia formation in hypotonic buffer was also confirmed by the analysis of the numbers of formed lamellipodia per cell, based on the CLSM observation of the cells stained with phalloidin (Supplementary Fig. 10c). These results also support the idea that M2[45–62] reduces cell membrane tension.

### Role of FBP17 in M2[45–62]-driven lamellipodium formation

Involvement of the FBP17 protein was investigated to confirm whether M2[45–62]-driven lamellipodium formation is induced by altered cell membrane tension. FBP17 has been reported to be a membrane tension sensor involved in lamellipodium formation^10^. FBP17 was distributed throughout the cell, forming dot- or patch-like assembled structures. However, once membrane tension was reduced, the protein was recruited to the cell membrane to activate WASP/N-WASP, thereby promoting Arp2/3 complex-dependent actin polymerization and co-localization with F-actin^10^ (Fig. 1b). Time-lapse live cell imaging was conducted to analyze the effect of M2[45–62] on the dynamics of FBP17 and F-actin using FBP17 fused to the green fluorescent protein (GFP-FBP17) and Lifeact-mCherry (Fig. 3b). No specific cellular localization of the signals was detected for either GFP-FBP17 or Lifeact-mCherry immediately after adding M2[45–62] (time 0). However, 12 min after adding M2[45–62], significantly polarized Lifeact-mCherry signals, suggestive of lamellipodium formation, were observed (Fig. 3b), in accordance with the results obtained by phalloidin staining (Fig. 2a). Marked accumulation of GFP–FBP17 signals at the periphery of the cell and co-localization with the Lifeact-mCherry signals were also observed (Fig. 3b). This result indicates the possible involvement of FBP17 in M2[45–62]-driven lamellipodium formation.

Involvement of FBP17 in M2[45–62]-driven lamellipodium formation was further confirmed using FBP17 knockdown cells. siRNA knockdown, analyzed by reverse transcription PCR and western blotting of cell lysates, reduced the FBP17 mRNA and endogenous protein levels in COS-1 cells to marginal levels (Supplementary Fig. 11). M2[45–62] yielded lamellipodia that were ~ 60% of control siRNA-treated cells. siRNA knockdown of FBP17 led to a decrease in the number of cells forming lamellipodia by 20% (Fig. 3d). These results strongly suggest that FBP17 is involved in M2[45–62]-driven lamellipodium formation, and that M2[45–62] induced lamellipodium formation by decreasing cell membrane tension. Note that membrane curvature has a close relationship with membrane tension. Asymmetric insertion of M2[45–62] may yields the decrease in cell membrane tension, accompanied by spontaneous curvature^35,36^. It has also been reported that FBP17 has a membrane curvature sensing ability^35^. Although further study is needed, recruitment of FBP17 to the cell membranes in accord with the M2[45–62]-driven spontaneous curvature formation may be involved in the mechanism of FBP17 in sensing membrane tension, leading to actin polymerization^39^.

### Lamellipodium formation ability of the M2[45–62] derivatives

**Fig. 4:**
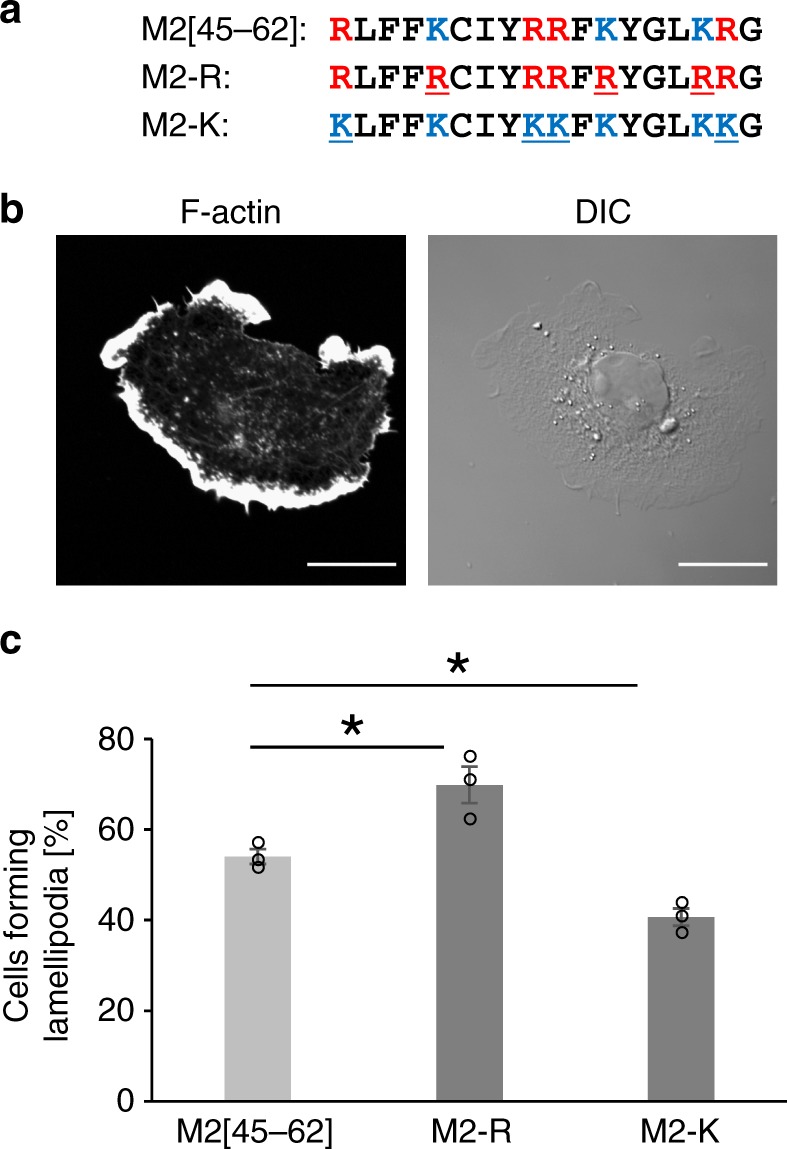
Arginine residues in M2[45–62] are more important for lamellipodium formation than are lysine residues. **a** Sequences of M2[45–62] and the M2-derivatives M2-R and M2-K. One-letter amino acid codes: R, arginine (Arg); L, leucine (Leu); F, phenylalanine (Phe); K, lysine (Lys); C, cysteine (Cys); I, isoleucine (Ile); Y, tyrosine (Tyr) and G, glycine (Gly). **b** Confocal laser scanning microscopy image of F-actin (stained with rhodamine–phalloidin) in COS-1 cells treated with 10 µm M2-R for 15 min. Scale bar, 20 µm. **c** Quantification of lamellipodium formation after treatment with M2[45–62] and the M2-derivatives (10 µm each). The mean ± standard error were derived from ~ 300 cells pooled from three independent experiments. *P < 0.05, Student’s *t* test

Basic amino acids including lysine (Lys) and arginine (Arg) often play important roles in peptide–membrane interactions^40^. The M2[45–62] sequence contains four Arg and three Lys residues. Substitution of these Arg and Lys residues may affect M2-driven lamellipodium formation. Therefore, we synthesized the M2[45–62]-derivatives M2-R, in which all Lys residues were replaced with Arg, and M2-K, in which all Arg residues were replaced with Lys (Fig. 4a), and evaluated the lamellipodium formation abilities of these peptides. M2-R exhibited higher (Fig. 4b), whereas M2-K showed lower, lamellipodium formation ability compared with M2[45–62] (Fig. 4c). Arg may have a more-important role in lamellipodium formation by M2[45–62] compared with Lys. The guanidino group in Arg forms bidentate hydrogen bonds with lipid phosphates, whereas Lys only forms a single hydrogen bond^40^. Therefore, Arg may more effectively interact with membranes, yielding more enhanced lamellipodia. The bidentate hydrogen bond formation propensity of Arg and the increased net positive charges in M2-R may also more favorably work for curvature formation than M2[45–62] and M2-K, as has been reported for arginine-rich cell-penetrating peptides^41^, leading to the reduction of cell membrane tension. On the other hand, the analysis using optical tweezers yielded no significant differences in tether force of M2-R from M2[45–62] (Supplementary Fig. 8). A subtle difference in cell membrane tension, which may be undetectable using an optical tweezers apparatus, can yield a significant difference in biological responses including lamellipodia formation.

### Suppression of cell migration by M2[45–62] and M2-R

**Fig. 5:**
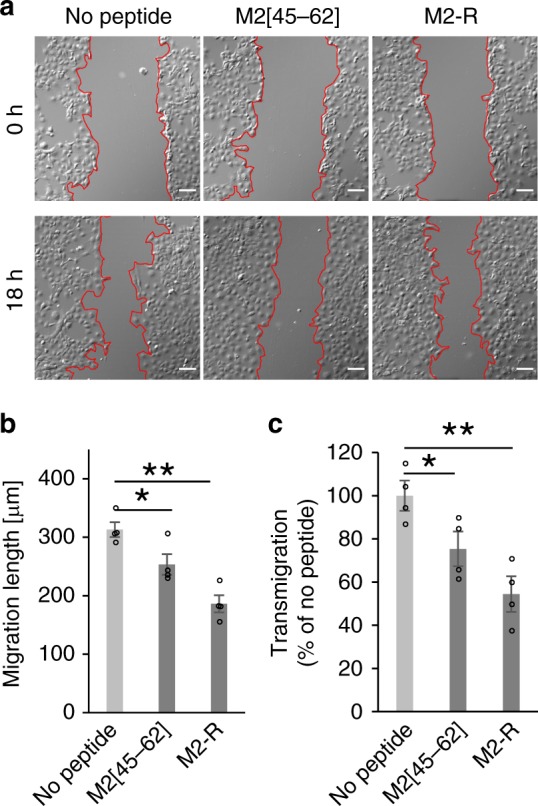
M2[45–62] and M2-R decreased cell migration. **a** Migration of COS-1 cells treated with M2[45–62] and M2-R (10 µm) for 18 h at 37 °C. Migration length was calculated by measuring the speed and efficiency with which cells in a monolayer migrate to close a gap in the monolayer. Scale bar, 100 µm. **b** Average migration length of COS-1 cells treated with M2[45–62] or M2-R (10 µm each) in serum-free medium for 18 h at 37 °C under 5% CO_2_, as described in the Supplementary Information. The average migration length during peptide treatment was calculated. **c** Relative transmigration of HeLa cells treated with M2[45–62] or M2-R (1 µm each) in serum-free medium for 6 h at 37 °C under 5% CO_2_, as described in the Supplementary Information. The cells that migrated to the lower chamber were stained with 3’,6’-di(*O*-acetyl)-4’,5’-bis[*N,N*-bis(carboxymethyl)aminomethyl]fluorescein, tetraacetoxymethyl ester (Calcein-AM). Relative signal intensities were calculated by comparing the fluorescence intensities of the peptide-treated and untreated cells. The fluorescence intensity was normalized to that of cells without peptide treatment. *P < 0.05, **P < 0.01, Student’s *t* test

Our results suggest that M2[45–62] can affect cell membrane tension. Thus, we investigated the effect of M2[45–62] on cell motility. The effect of M2-R was also evaluated, with the expectation that this peptide has higher activity than that of M2[45–62]. The scratch wound migration assay^42^ is a representative assay to analyze cell mobility. In this assay, the cell monolayer is scratched with a pipet tip to yield an area where cells are removed (i.e., wound, Fig. 5a, band areas indicated with the red lines in the center of the pictures). The migration of cells yields invasion of the cells into the band area. Cell migration efficacy is thus assessed as the extent of the wound closure (i.e., decrease in the band width) using CLSM (Fig. 5a). After scratching the monolayer, COS-1 cells were treated with the peptide (M2[45–62] or M2-R) for 18 h. Wound closure (or mobility) of cells treated with M2[45–62] and M2-R was decreased markedly compared with that of untreated control cells (Fig. 5b). The migration distances of the cells treated with M2[45–62] and M2-R were 83% and 60% of the untreated cells, respectively. Therefore, these peptides reduced cell mobility. No significant cytotoxicity was observed in the cells treated with either M2[45–62] or M2-R under the assay conditions (Supplementary Fig. 12).

The effect of these peptides on cell mobility was further evaluated by an in vitro transwell migration assay^43^, as described in the Supplementary Information. In brief, HeLa cells were placed in the upper chamber with a permeable membrane. The extent of cell migration was evaluated by counting the cells that migrated into the lower compartment through the membrane pores. The proportions of cells treated with M2[45–62] and M2-R that migrated to the lower compartment were 74% and 54% of the untreated cells that migrated, respectively (Fig. 5c). Therefore, M2[45–62] and M2-R significantly impaired the ability of HeLa cells to migrate toward the lower compartment. These results coincide with previous reports that a decrease in membrane tension is correlated with a decrease in cell motility by unorganized, rough lamellipodium formation^7–9^.

### Effects of M2[45–62] on neuronal morphology

**Fig. 6:**
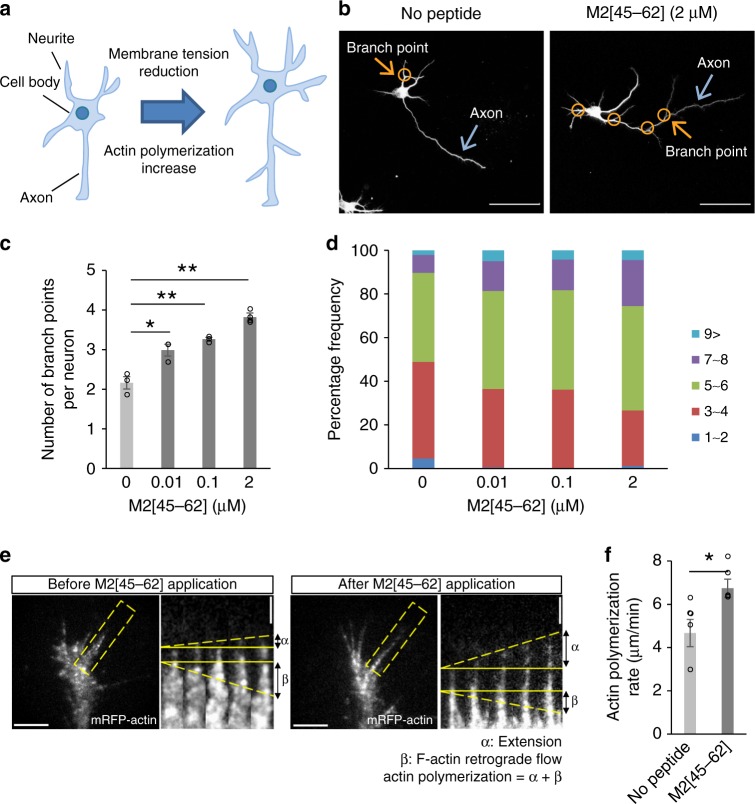
M2[45–62] increases neurite outgrowth in cultured neurons. **a** Possible effects of cell membrane tension on neuronal morphology. **b** Confocal laser scanning microscopy image of neuronal morphology (stained with anti-Tuj1 antibody) after treatment with M2[45–62]. Scale bar, 50 µm. **c** Quantification of the average number of branch points per neuron of the control and M2[45–62]-treated neurons. The mean ± standard error were derived from ~ 150 neurons pooled from three independent experiments. *P < 0.05, **P < 0.01, Student’s *t* test. **d** Distribution of neurite number in cells treated with M2[45–62]. The number of neurites per cell was counted from microscopy images, and the percentages of the neurons having the said numbers of neurites were obtained. The means were derived from ~ 150 neurons pooled across three independent experiments. An increase in M2[45–62] concentration resulted in an upper shift in the median distribution. **e** Fluorescent speckle microscopy images of mRFP-actin in a growth cone in cultured neurons before and after M2[45–62] administration (scale bar, 5 µm). Kymographs (space–time plots displaying intensity values along a predefined path over time) of the flow of fluorescent features of mRFP-actin in filopodia at 5-s intervals are shown (scale bar, 2 µm). F-actin extension and retrograde flow are indicated by dashed yellow lines. The sum of F-actin extension and retrograde flow (a + b) indicates the rate of mRFP-actin turnover, which reflects the rate of actin polymerization^42^. **f** The rate of actin polymerization in a growth cone of neurons before and after applying M2[45–62]. In total, 27 cells (no peptide = PBS treatment) and 26 cells (M2[45–62] treatment) were analyzed. *P < 0.05, Student’s *t* test

Awareness of the importance of neuronal morphology in brain function has increased^44^. As demonstrated above, changes in membrane tension can lead to actin reorganization, which can alter cellular morphology. A neuron is comprised of the cell body and neurites (Fig. 6a). Neurites are projections from the cell body that are involved in communication with other neurons. The cell body usually has a single long neurite called the axon. As actin contributes to the characteristic structures of neuronal cells, the potential effects of M2[45–62] on cell membrane tension and thus actin polymerization may be more apparent in these cells. Thus, the effects of M2[45–62] on neuronal morphology were assessed.

Primary cultured rat hippocampal neurons were treated with M2[45–62] (0.001, 0.01, 0.1, or 2 µm) for 48 h. Neuronal morphology was assessed by staining the neurons with an anti-Tuj1 (neuronal class III beta-tubulin) antibody and viewing the stained neurons under a microscope (Fig. 6b). Neuronal morphology is described by the axon length, total neurite length, and the number of neurites (Fig. 6d, Supplementary Fig. 13). Branching of neurites is also an important factor determining neuronal morphology^45^, and this was evaluated by the number of branch points per neuron (Fig. 6c).

Neurons treated with 2 µm M2[45–62] had a significantly greater axon length (~ 15%) and total neurite length (~ 30%) (Supplementary Fig. 13a, b). A more-marked effect was observed on the number of branch points per neuron. A significant dose-dependent increase in the number of branch points was observed in the M2[45–62]-treated cells (Fig. 6c). Untreated cells had an average of two branch points, whereas cells treated with 2 µm M2[45–62] had double the number of branch points. The M2[45–62] treatment tended to increase the number of neurites per neuron (Fig. 6d).

These results clearly showed the ability of M2[45–62] to stimulate neurite outgrowth, which may accompany the decrease in cell membrane tension induced by the peptide and the eventual decrease in the tension barrier for actin polymerization. The increase in the rate of F-actin polymerization induced by M2[45–62] was further confirmed by analyzing actin extension and retrograde flow in growth cones using fluorescent speckle imaging^46^ (Fig. 6e and Supplementary Movies 1 and 2). Fluorescent speckle imaging enables the detection of molecules attached to F-actin that flow like moving speckles or distinct puncta against a low fluorescence background^47^. Monomeric red fluorescent protein-tagged actin (mRFP-actin) was employed to visualize F-actin retrograde flow. A growth cone represents the growing end of an extending neurite and is characterized by active actin polymerization. The filopodium in a growth cone, which is highlighted in the dashed rectangles in Fig. 6e, is a narrow cylindrical extension in which the attachment of globular actin (G-actin) to F-actin leads to F-actin extension. The extension of F-actin proceeds in the direction of the cell body by cellular myosin motors (i.e., retrograde F-actin flow). Therefore, the rate of actin polymerization is defined as the sum of F-actin extension and retrograde flow.

A 1.4-fold higher rate of actin polymerization was observed in growth cones of neurons treated with M2[45–62] (2 µm) compared with untreated neurons (Fig. 6f). These results strongly suggest that M2[45–62] stimulates actin polymerization and increases neurite outgrowth in rat primary hippocampal neurons by reducing cell membrane tension. The potential applicability of growth factors, such as neurotrophins, for treating inflammatory damage in the central nervous system (CNS) has been considered^48^. Thus, changing cell membrane tension and increasing neurite outgrowth may provide a new approach for treating CNS disorders.

## Discussion

**Fig. 7:**
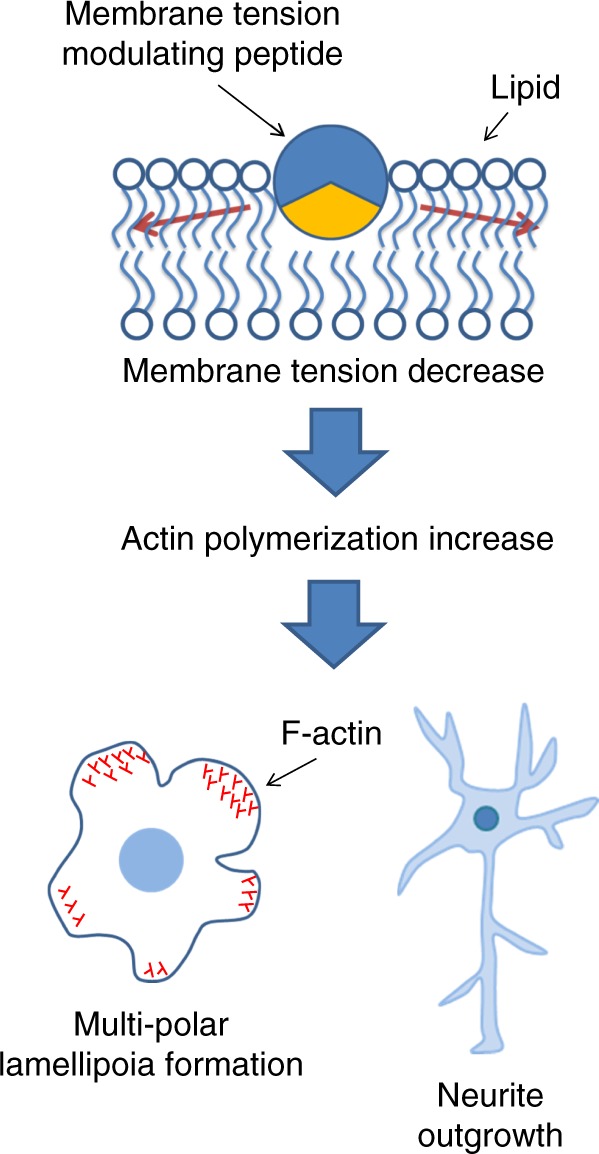
Outline of this study. Membrane interaction of M2[45–62] leads to decrease in cell membrane tension. FBP17-mediated actin polymerization is then induced, resulting in suppression of cell migration via multipolar lamellipodia formation (COS-1) and neurite outgrowth (cultured neuron)

Although cell membrane tension is considered to play a critical role in regulating cell movement and morphology, few approaches have been realized to develop chemical tools to regulate cell movement and morphology via focusing membrane tension. In this study, we focused on peptides corresponding to the amphipathic segments derived from proteins with membrane deforming activity (Fig. 7). The M2[45–62] peptide derived from the influenza M2 protein exhibited actin polymerization and lamellipodium formation at multiple sites in the cells, suggesting that inserting M2[45–62] reduced cell membrane tension. The effect of M2[45–62] on cell membrane tension was confirmed by an optical tweezers experiment and a gating analysis of mechanosensitive channels. Involvement of the membrane tension sensor FBP17 was also suggested in this step. Importance of Arg in M2[45–62] membrane interactions was exemplified by substituting Lys with Arg in M2[45–62]. Lamellipodium formation in multiple directions by M2[45–62] suppressed cell movement. Actin polymerization by M2[45–62] resulted in neurite outgrowth with increased neurite branching in primary cultured rat hippocampal neurons. Considering membrane tension has a close relationship with membrane curvature, further exploration is needed of the contribution of membrane curvature to the M2[45–62]-mediated reduction in cell membrane tension and the accompanying cellular events.

We have demonstrated the possibility and promise of modulating cell movement and morphology by externally manipulating cell membrane tension using peptides. These features may also be applicable to studies that involve cell migration and invasion, including those of cancer cells. These tools also have therapeutic potential for preventing cell migration and restoring nerve cell injury in CNS diseases. Of course, further investigation is needed to identify the details of the mechanism of cell membrane tension modulation by M2[45–62], referring other cellular factors including e.g., curvature. However, we believe that this report opens new doors for cell manipulation using molecular tools by focusing membrane tension.

## Methods

### Materials

All reagents including salts and incubation media were obtained from Sigma-Aldrich and Wako unless otherwise specified. Polyclonal anti-FBP17 (1:200 for immunoblotting; sc82149, Santa Cruz Biotechnology) was purchased. The following constructs have been described previously: human GFP–FBP17^49^ and Lifeact-mCherry^50^.

### Peptides

Peptides were synthesized using Fmoc-solid-phase synthesis on a Rink amide resin, as reported previously^51^. The NBD-labeled peptides were prepared on resin by the treatment of the N-terminal amino group with 4-fluoro-7-nitrobenz-2-oxa-1,3-diazole as previously described^52^. The peptides was then deprotected and cleaved from the resin by treatment with a trifluoroacetic acid–ethanedithiol mixture (95:5) at room temperature (~ 20 °C) for 3 h, followed by lyophilization. Purification of the desired peptides on reverse-phase high-performance liquid chromatography (RP-HPLC) followed by lyophilization of the eluent yielded the pure peptides. Purity and identity of the respective peptides were confirmed by RP-HPLC and matrix-assisted laser desorption-ionization time-of-flight mass spectrometry (Supporting Information Table S2).

### Preparation of LUVs

1-Palmitoyl-2-oleoyl-*sn*-glycero-3-phosphocholine (POPC) and 1-palmitoyl-2-oleoyl-*sn*-glycero-3-phospho-(1’-*rac*-glycerol) (POPG) were obtained from NOF Corporation. For NBD binding assays, a lipid film was formed by rotary evaporation of a mixture of POPC/POPG/Chol (molar ratio = 4:1:2) in chloroform. After vacuum-drying overnight, the lipid film was hydrated with phosphate buffered saline (PBS: 137 mm NaCl, 2.7 mm KCl, 10 mm Na_2_HPO_4_, and 1.8 mm KH_2_PO_4_. LUVs were generated through 21 successive extrusions of the lipid dispersions through double polycarbonate filters with 100-nm pore size, using a Liposofast extrusion system (Avestin). The concentration of LUVs was determined using a LabAssay Phospholipid kit (Wako) and expressed as the lipid concentration.

### Cell culture, transfection, and RNAi

COS-1 cells were cultured in Dulbecco’s Modified Eagle Medium (DMEM) supplemented with 10% heat-inactivated fetal bovine serum. Transfection was carried out using Lipofectamine LTX Reagent (Invitrogen) according to the manufacturer’s protocol. Transfected cells were examined after 24 h. Note that overexpressed F-BAR proteins tend to form aggregated tubules of cell membrane and thus cannot be polarized. Therefore, we chose cells with low expression. For knockdown experiments, Dharmacon SMARTpool-ON-TARGETplus siRNAs (GE Healthcare) against human FBP17 were used. ON-TARGETplus Non-Targeting siRNA Pool (GE Healthcare) was used as control siRNA. RNAs (20 nm) were transfected into cells with Lipofectamine 3000 (Invitrogen). Cells were then cultured for 72 h and used for analysis. We confirmed the knockdown of FBP17 by RT-PCR and western blotting (Supplementary Fig. 9).

Hippocampal neurons prepared from embryonic day 18 rat embryos were cultured on glass coverslips (Matsunami) coated with polylysine, as described previously^47^. For Fluorescent speckle imaging, neurons were transfected with plasmid DNA using Nucleofector (Lonza) before plating.

### NBD binding assay

NBD-labeled peptides were tested at a concentration of 500 nm in PBS treated with each concentration of LUVs at room temperature. The fluorescence intensity was measured at an excitation wavelength of 485 nm and emission of 535 nm using a Wallac 1420 Victor2 Microplate Reader (PerkinElmer). To correct for background, the readings obtained when buffer was titrated with lipid vesicles were subtracted from each recording of fluorescence intensity.

### Confocal microscopy

For F-actin observation, COS-1 cells were plated into 35-mm glass-bottomed dishes (Iwaki) and incubated for 4.5 h. Then cells were treated with each amphipathic peptide in isotonic buffer (20 mm HEPES at pH 7.5 containing 150 mm NaCl, 5 mm KCl, 1 mm CaCl_2_, 1 mm MgCl_2_, and 10 mm glucose) for 15 min, fixed with 4% paraformaldehyde in PBS for 10 min, and permeabilized with 0.1% Triton X-100 for 4 min prior to staining with rhodamine–phalloidin (Invitrogen) for 30 min. Fluorescence images were captured using a confocal microscopy system through an objective lens (× 60 oil immersion objective, numerical aperture = 1.35). After confocal microscopy observation, each cell was evaluated whether or not lamellpodia was formed.

For live cell imaging, transfected COS-1 cells were grown on 35 mm glass-based dishes. Just before imaging, the culture medium was changed to isotonic buffer supplemented with 10% fetal bovine serum (volume, 150 µL). The cells were placed at 37 °C in a microchamber (MI-IBC, Olympus) attached on the stage of the inverted microscope. M2[45–62] dissolved in isotonic buffer supplemented with 10% fetal bovine serum (volume, 50 µL) was added to the cell moderately co-expressing GFP–FBP17 and Lifeact-mCherry to yield final peptide concentration of 20 μm, and images were acquired every 1 min using a confocal microscope (FV1000, Olympus).

For hypotonic shock treatment, COS-1 cells were treated with M2[45–62] (5 µm) in isotonic buffer as described in live cell imaging. COS-1 cells were then treated with an equal volume of hypotonic buffer (1 mm CaCl_2_ and 1 mm MgCl_2_; volume, 200 µL) immediately after M2[45–62] treatment. And images were acquired as described above.

For observing NBD-labeled peptides, COS-1 cells were treated with NBD-labeled peptides (5 µm) for 15 min and analyzed by CLSM. After observing NBD fluorescence distribution, dithionite (20 µm) was added and CLSM observation was conducted 1 min later.

### Scratch wound migration assay

COS-1 cells (35 mm glass-bottomed dish, Iwaki) were washed with DMEM without containing serum [DMEM(–)] and incubated with the medium for 6 h at 37 °C under 5% CO_2_ before the scratch wound was made with a 10 µL-pipette tip. The cells were treated with M2[45–62] or M2-R (10 µm each) in DMEM(–) (1 mL) for 18 h at 37 °C under 5% CO_2_. The cells were observed by FV1000 confocal scanning laser microscope (Olympus) equipped with a × 10 objective before and after treatment of the peptides. Migration of cells was analyzed using ImageJ 1.50i (NIH, Bethesda, MD). Migration length was calculated by comparing average distance between area surrounded by red lines before peptide treatment and after 18 h incubation. No peptide represents the cells similarly incubated in DMEM(–) without containing peptides.

### Transwell migration assay

HeLa cells were released from plates and then resuspended in serum-free α- minimum essential media (α-MEM(–)). The cells (2 × 10^4^ cells) were replated onto the upper chamber of a Transwell filter (Costar; 8-μm pore size). The cultured medium containing serum was added to the lower chamber. After 6 h of incubation at 37 °C, transwells were removed from the plates and cells in the bottom wells were stained with calcein-AM (Dojindo). The fluorescence intensity in each well was determined using a Wallac 1420 Victor2 Microplate Reader (PerkinElmer). No peptide represents the fluorescence intensity of the wells for the cell similarly incubated in α-MEM(–) without containing peptides.

### Cell viability assay

Cell viability was determined using Cell Proliferation Reagent WST-1 (4-[3-(4-lodophenyl)-2-(4-nitrophenyl)-2H-5-tetrazolio]-1,3-benzene disulfonate) (Roche), following the manufacturer’s protocol. In brief, COS-1 cells were treated with peptides at various concentrations for 30 min in isotonic buffer. WST-1 was then added, and the cells incubated for another 1 h. No peptide represents the viability of the cells similarly incubated in isotonic buffer without containing peptides.

As for the experiments in Supplementary Fig. 10, COS-1 cells were treated in same condition as wound healing assay. Cell viability was analyzed using Cell Counting Kit-8 (CCK8) (Dojindo), according to the manufacturer’s protocol.

### Fluorescent speckle imaging

Fluorescent speckle imaging and speckle tracking analysis were performed as described previously^47^. The rate of actin polymerization was calculated as the sum of F-actin extension and retrograde flow^46^. No peptide represents rate of actin polymerization of the cells similarly incubated in Neurobasal medium without containing peptides.

### Circular dichroism

CD spectra were recorded using a Jasco 820 UV–vis spectropolarimeter. Spectra were recorded over a wavelength range of 200–260 nm with a spectral bandwidth of 1 nm, a time response of 0.5 s, a scan speed of 50 nm/min, and a step resolution of 0.5 nm. Each spectrum was expressed over an average of five spectra. Spectra were measured for a 10 μM peptide solution dissolved in isotonic buffer containing 50% trifluoroethanol at 25 °C. The molar ellipticity was expressed per decimal residue.

### Tether force measurements by optical tweezers

The cells were observed with bright field using an optical tweezers system (NanoTrackerTM2, JPK Instruments) equipped with an infrared laser source (3W, 1064 nm) on an Olympus IX-73 inverted microscope. A silica bead (1.5 µm, Polysciences) was attached to the cell membrane and was pulled away to form a membrane tether (5 µm length) by moving the microscope stage under computer control at 1 µm s^−1^. Tether force (*F*_*tether*_) can be calculated using Hooke’s law: *F*_*tether*_ = kΔ*x* (k: trap stiffness, Δ*x*: the displacement of the bead from the center of the trap). Trap stiffness (k, typically ~ 0.15 pN/nm) was calibrated for each experiment by a power spectrum analysis^53^.

### Electrophysiology

*Escherichia coli* strain PB104 (*ΔMscL*::*Cm*) was used as host for the pB10 expression constructs^54^. *E. coli* spheroplasts were prepared as described previously^55^ and the channel activity of MscS and MscL were examined by the inside-out patch-clamp method^56^. The gating threshold of MscS and MscL are the pressure at which the first channel opening was observed. Negative pressure was applied by syringe-generated suction through the patch pipette and measured with a pressure gauge (PM 015R, World Precision Instruments, Sarasota, FL). Pipette solutions contained 200 mm KCl, 90 mm MgCl_2_, 10 mm CaCl_2_, and 5 mm HEPES (pH 6.0), whereas the bath solution additionally contained 0.3 m sucrose. The pipette potential was held at + 20 mV relative to the bath. Currents were amplified using an Axopatch 200B amplifier (Axon Instruments, Foster City, CA) and filtered at 2 kHz. Current recordings were digitized at 5 kHz using a Digidata 1322A interface with pCLAMP 10 software (Axon Instruments, Foster City, CA).

### Statistics and reproducibility

Statistical analysis was carried out using a two tailed Student *t* test. *P* values were determined with Microsoft Excel and GraphPad Prism 6. Differences were considered significant when the calculated *p* value was < 0.05. *P* values are described in figure legends. All experiments were successfully replicated. The number of replicates is specified for each experiment.

### Reporting summary

Further information on research design is available in the Nature Research Reporting Summary linked to this article.

## Supplementary information

Supplementary Information

Supplementary Data 1

Reporting Summary

Supplementary Movie 1

Supplementary Movie 2

Description of Additional Supplementary Files

## Data Availability

The data that support the findings of this study are available from the corresponding author on reasonable request.
