# Supplementary material for: An influenza-derived membrane tension-modulating peptide regulates cell movement and morphology via actin remodeling
Source: Commun Biol. 2019 Jun 26;2:243. doi: 10.1038/s42003-019-0486-3 (PMC6594980; doi:10.1038/s42003-019-0486-3)
Supplement: Supplementary file 1 — Supplementary Information [file 42003_2019_486_MOESM1_ESM.pdf]

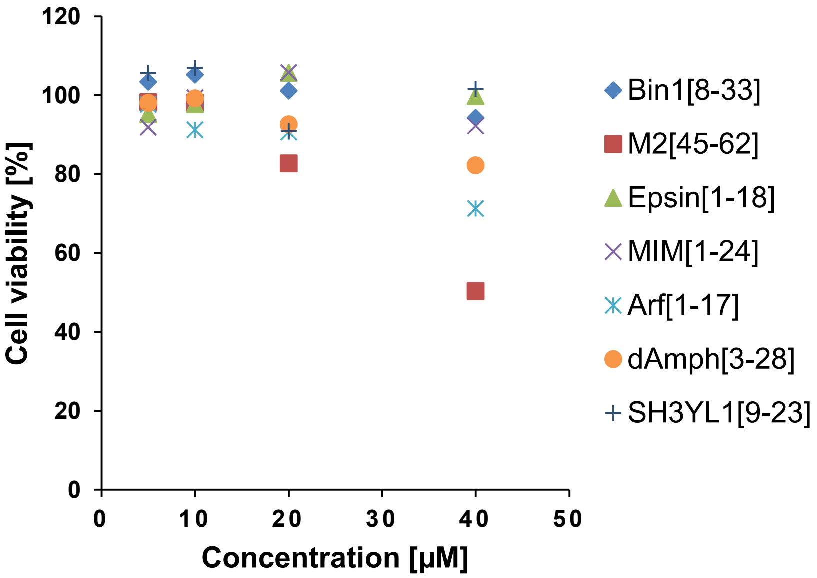

Supplementary Figure 1. Evaluation of the cytotoxic effects of the amphipathic peptides (WST-1 assay). COS-1 cells were treated with the peptides for 30 min and then subjected to the WST-1 assay. Results are presented as means ± standard error (n = 3).

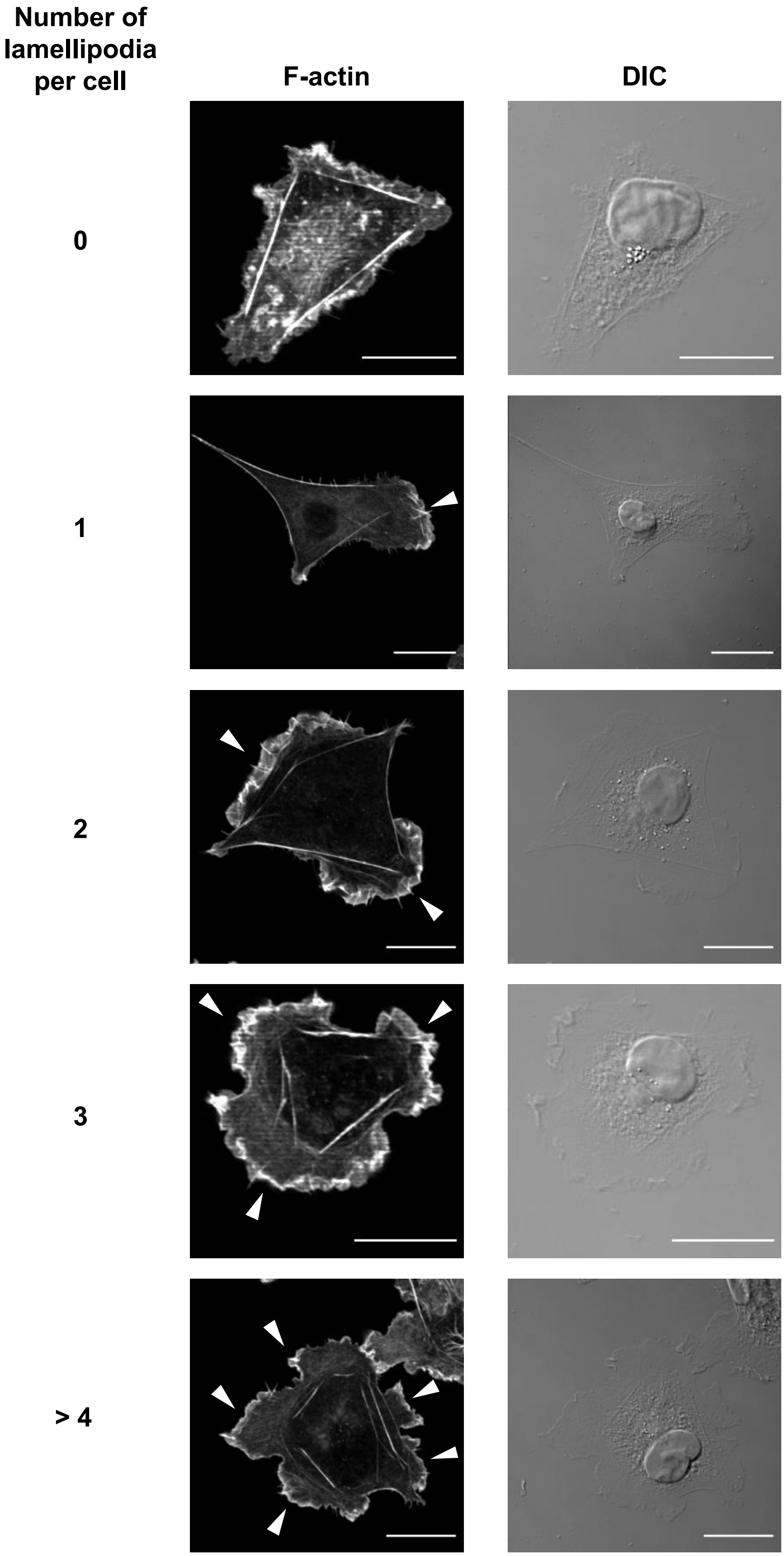

Supplementary Figure 2. Representative images of M2 treated cells bearing different numbers of lamellipodia (scale bars, 20 μm).

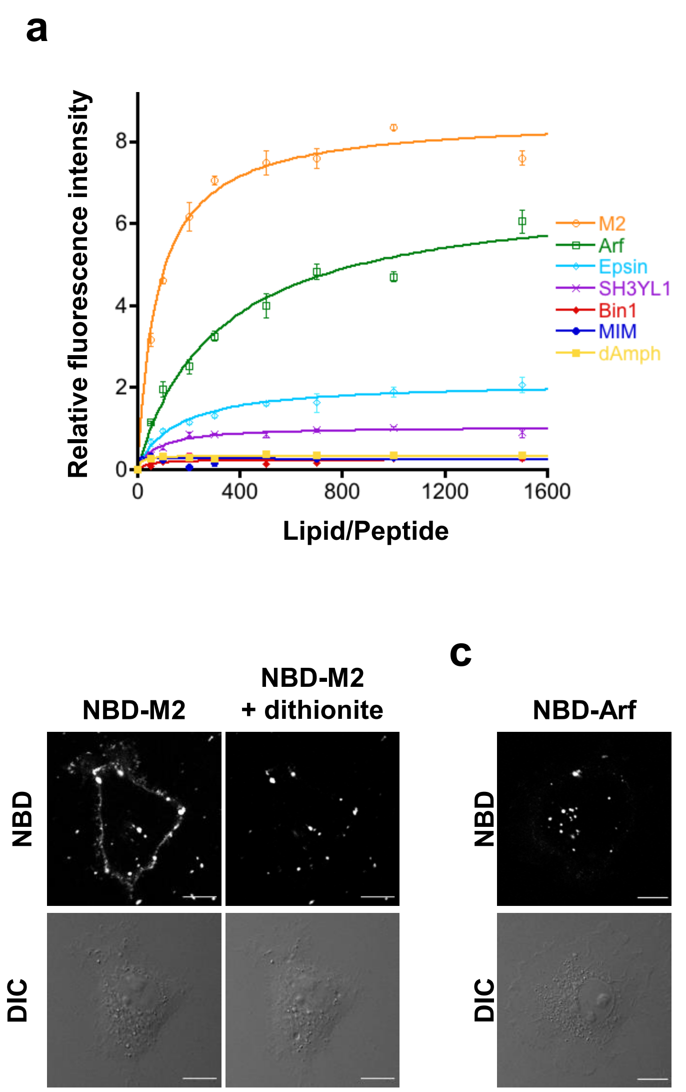

Supplementary Figure 3. a, Increased fluorescence of NBD-labelled peptides upon titration with liposomes. NBD-labelled peptides (0.5 µM) were titrated with liposomes with an excitation wavelength of 485 nm and emission wavelength of 535 nm. **b,** Confocal laser scanning microscopy (CLSM) images of NBD fluorescence in COS-1 cells treated with NBD-M2 (5 µM) for 15 min. After the NBD-M2 treatment, dithionite (20 µM) was added, and the samples were observed by CLSM 1 min later. Disappearance of the NBD-M2 signal along the perimeters of the cells suggested cell surface localization of the peptide. Scale bars, 10 µm. **c,** Confocal microscopy images of NBD fluorescence in COS-1 cells similarly treated with NBD-Arf (5 µM) (scale bars, 10 µm)..

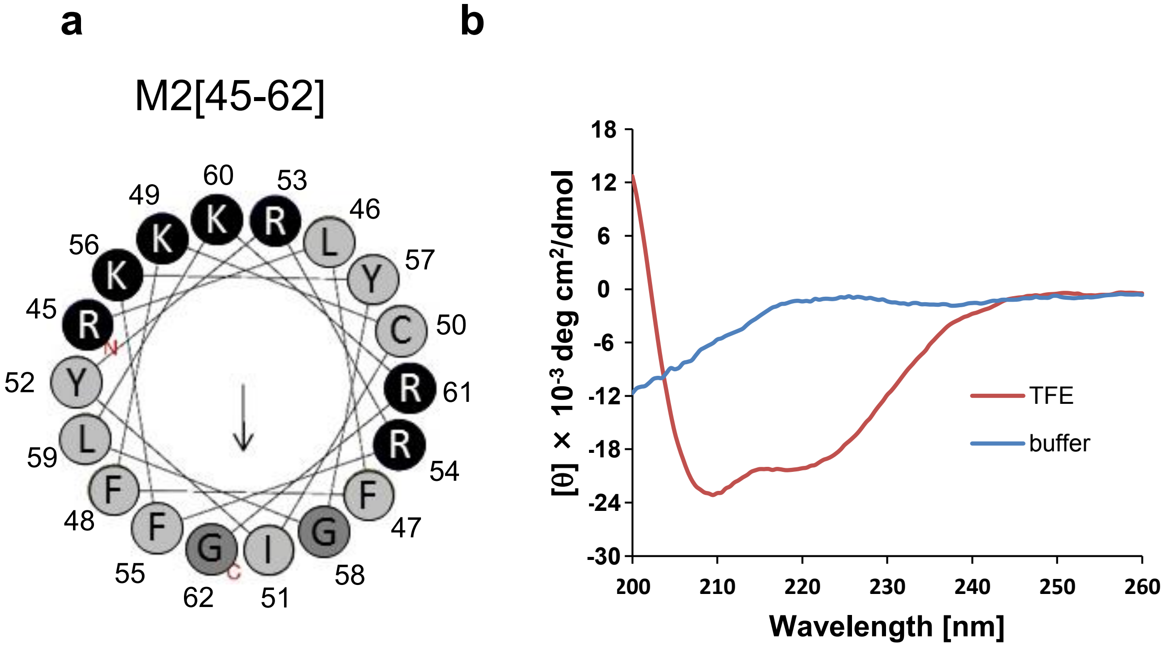

Supplementary Figure 4. M2[45–62] forms an amphipathic helical structure. **a,** Helical wheel plot of the M2[45–62] amphipathic helix is shown as generated using HeliQuest (http://heliquest.ipmc.cnrs.fr/). One-letter amino acid codes are given in the legend of Fig. 4. Hydrophilic residues are shown in black, Gly in dark grey, and hydrophobic resides in grey. N and C in red denote the N- and C-termini of the peptide, respectively. An arrow in the helical wheel represents the mean amphipathic moment, <μH>, given by HeliQuest based on the procedure described by Eisenberg, D., Weiss, R.M., & Terwilliger, T.C. *Nature* **299**, 371–374 (1982). **b,** CD spectra of M2[45–62] in PBS (blue) or in PBS containing 50% trifluoroethanol (TFE) (red) (peptide concentration of 10 μM).

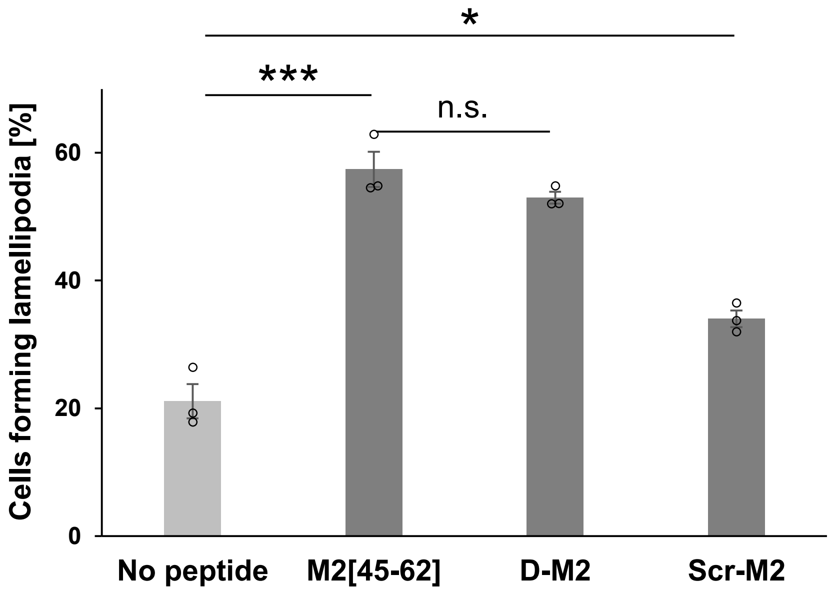

Supplementary Figure 5. Quantification of lamellipodium formation by M2[45–62], D-M2 and Scr-M2 (10 μM) treatments. The means ± standard error were derived from ~350 cells pooled from three independent experiments. *P < 0.05 ***P < 0.001, Student's *t*-test.

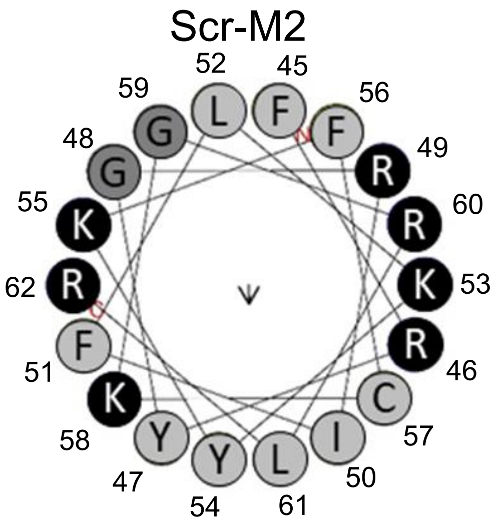

Supplementary Figure 6. Helical wheel plot of the Scr-M2 sequence (FRYGRIFLKYKFCKGRLR-amide) generated using HeliQuest (http://heliquest.ipmc.cnrs.fr/). This peptide was designed such that the hydrophobic amino acids in M2[45–62] were less clustered on one side of the helix (and thus less amphiphilic). Hydrophilic residues are shown in black, Gly in dark grey, and hydrophobic residues in grey. Red N and C denote the N- and the C-termini of the peptide, respectively. The N-terminal amino acid F was assigned residue number 45, and the C-terminal R residue was assigned number 62, for ease of comparison to M2[45–62]. The arrow in the helical wheel indicates the hydrophobic moment given by HeliQuest (see Supplementary Figure 2).

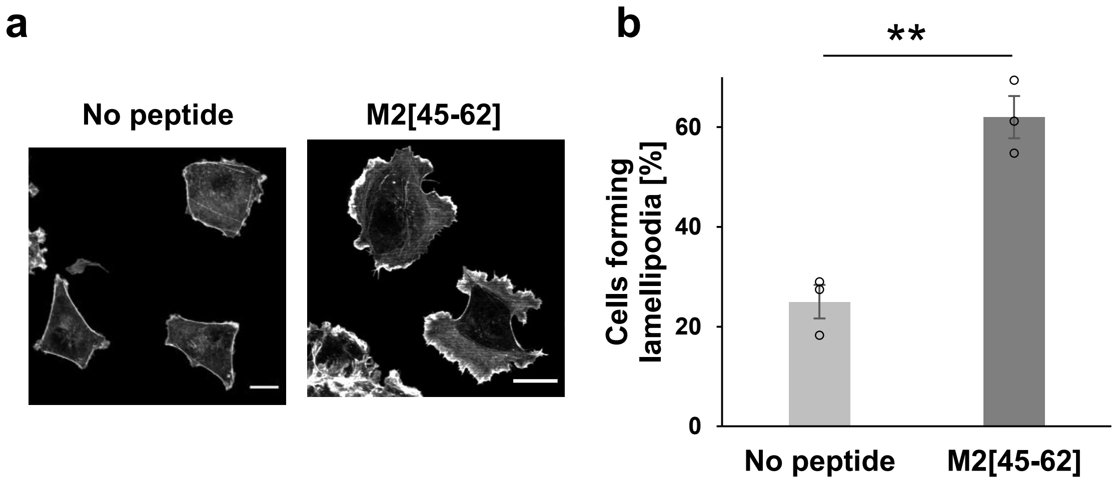

Supplementary Figure 7. M2[45–62] induced lamellipodium formation in the presence of serum. **a**, Confocal laser scanning microscopy images of F-actin (stained with rhodamine–phalloidin) in COS-1 cells treated with M2[45–62] for 15 min in the presence of serum. Scale bars, 20 μm. **b**, Quantification of lamellipodium formation induced by M2[45–62] in the presence of serum. The means ± standard error were derived from ~300 cells pooled from three independent experiments. **P < 0.01, Student's *t*-test.

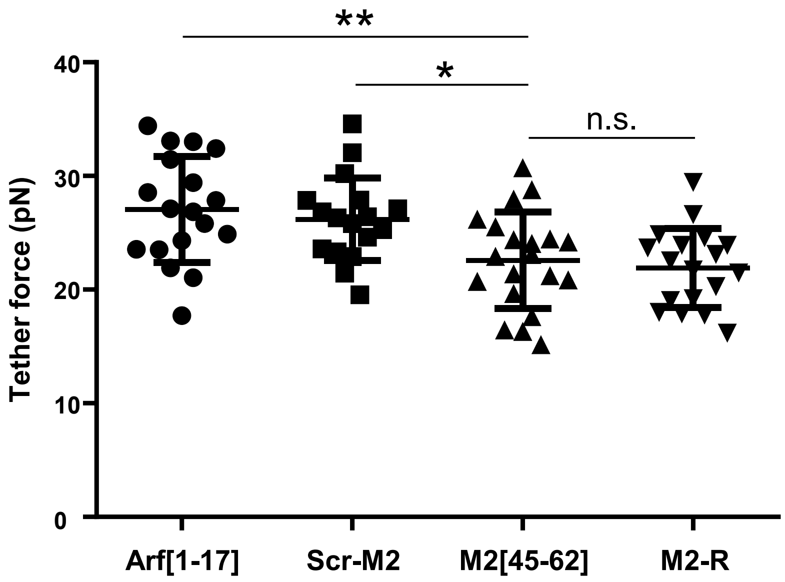

Supplementary Figure 8. Measurement of the tether force in COS-1 cells. The tether force of identical cells was measured after treatment with each peptide (20 μM). The mean ± standard deviation were derived from data pooled from three independent experiments. In total, 18 cells (Arf[1-18], Scr-M2 and M2-R) and 20 cells (M2[45–62] treatment) were analyzed. *P < 0.05 ***P < 0.001, one-way ANOVA Tukey's test.

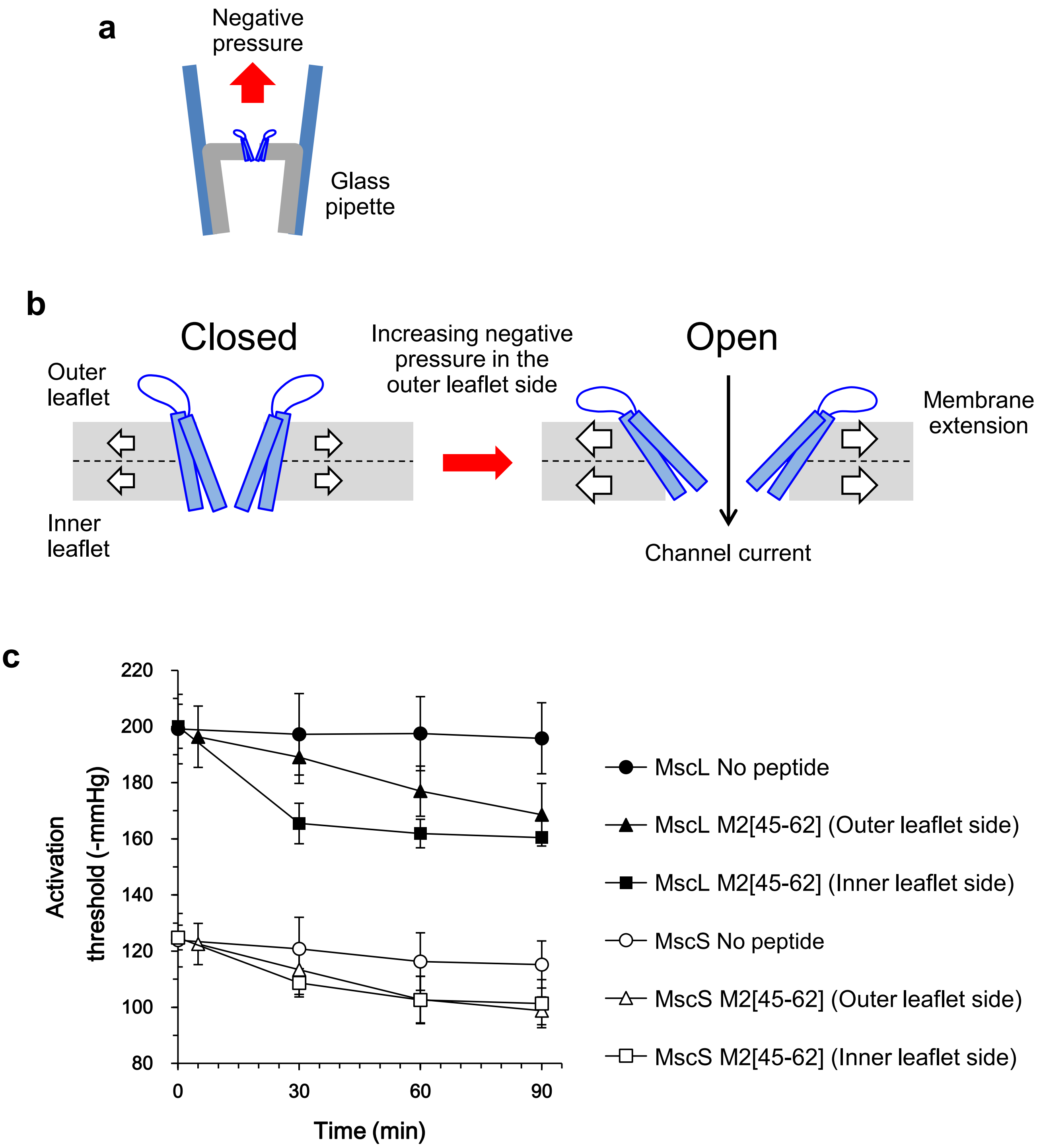

Supplementary Figure 9. Effect of M2[45–62] on the opening of mechanosensitive channels. **a**, Schematic representation of the analytical system used. A patch pipette was attached on the membrane surface, followed by a gentle suction in the pipette to form the gigaseal. The gigaseal is a tight attachment of the membrane surface to inner wall of the pipette without allowing ions to pass between the pipette and the membrane. An excised patch was formed by tearing off the patch from the bacterial cell membrane, where the suction was released to establish zero transmural pressure across the patch. Under this configuration, suction in the pipette leads to an extension of the patch membrane surrounded by the gigaseal. **b**, Extended membranes leading to open channels. **c**, Threshold pressures for activation of MscS and MscL in the presence of M2[45–62] compared with control conditions. M2[45–62] was added from either the outer or inner leaflet side. Threshold pressures needed to open these channels were plotted at 5, 30, 60 and 90 min after adding 40 μM M2[45–62]. The time dependent effects of the peptide suggest that stable distribution of the peptide requires time-consuming partitioning into the membrane. Note the asymmetric time dependency typically observed in the case of MscL, which suggests that the peptide primarily affects from inner leaflet of the bilayer in this setup.

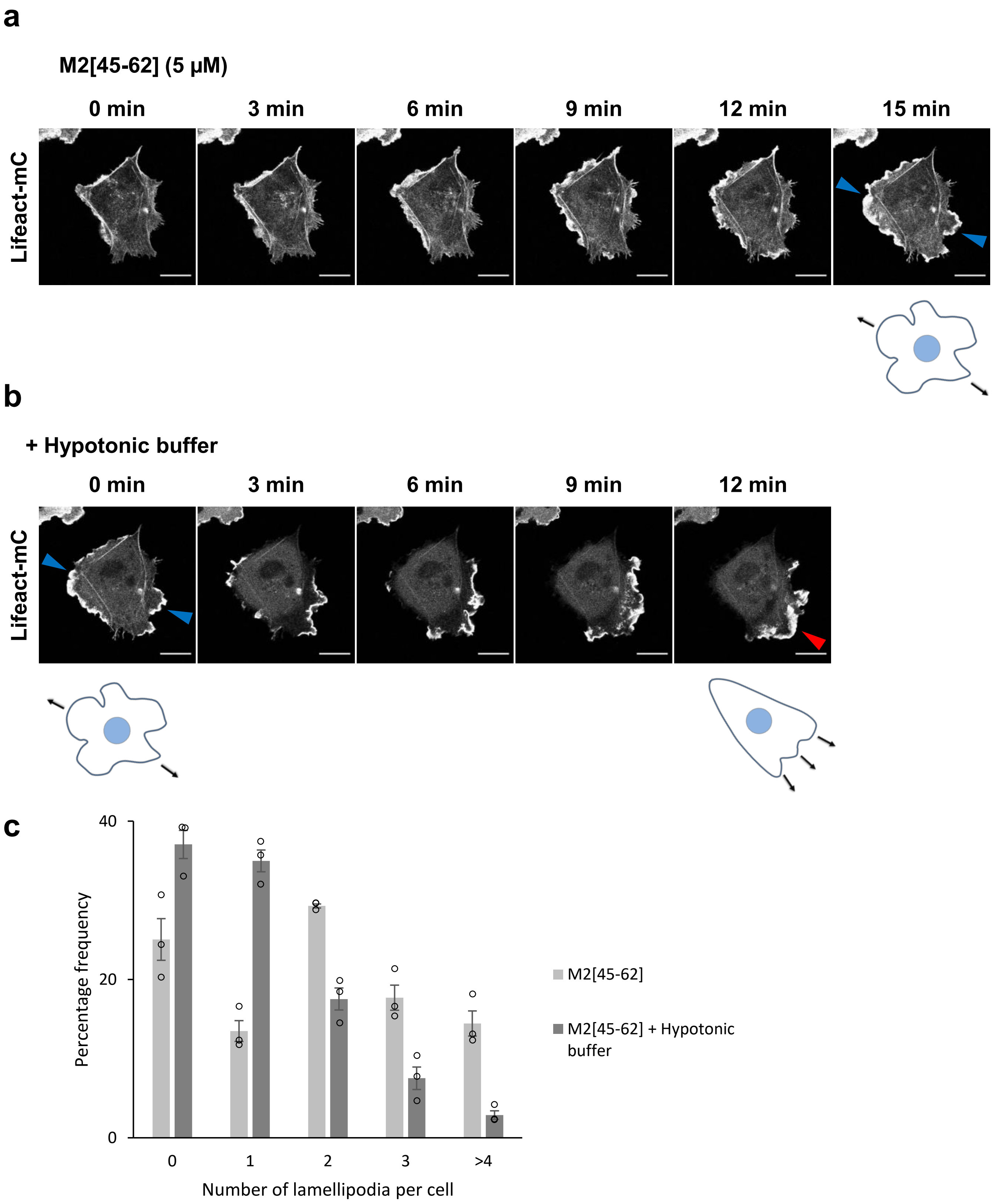

Supplementary Figure 10. Hypotonic shock resulted in the integration of lamellipodia from multiple sites into one main site. Time-lapse images of COS-1 cells expressing Lifeact–mCherry after adding M2[45–62] (5 μM) (**a**) and hypotonic buffer at 15 min after M2[45–62] treatment (**b**). COS-1 cells were treated with M2[45–62] and observed for 15 min. An equal volume of hypotonic buffer was added to a glass-based dish, and the COS-1 cells were observed for another 12 min. Scale bars, 20 μm. Blue arrowheads indicate lamellipodia at multiple sites. The red arrowhead indicates one main lamellipodium. The cartoons in (**a**) and (**b**) correspond to the cellular states in Fig. 1a. **c,** Distribution of the number of lamellipodia per cell after treatment with M2[45–62] and following hypotonic buffer, based on the CLSM observation of the cells stained with phalloidin. The means ± standard error were derived from ~300 cells pooled from four independent experiments.

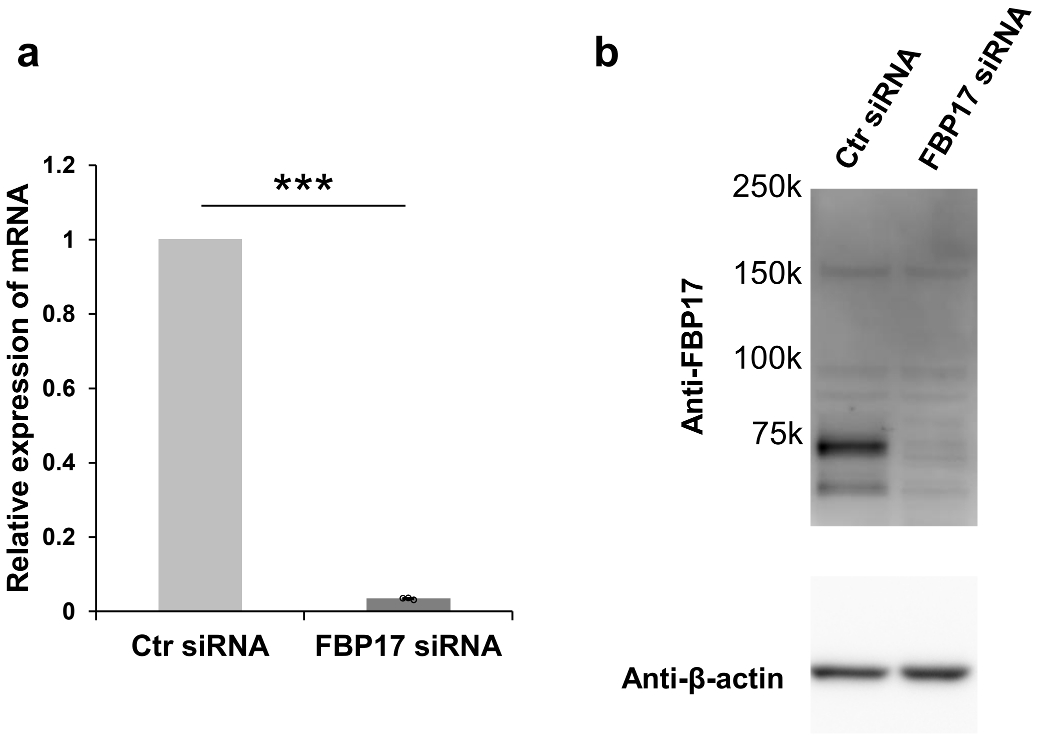

Supplementary Figure 11. Confirmation of the effect of siRNA on FBP17 expression. **a,** FBP17 mRNA (**a**) and protein (**b**) levels in COS-1 cells transfected with FBP17 siRNA were analyzed by reverse transcription PCR and western blotting using a specific antibody against FBP17, respectively. ***$P < 0.001$, Student's t-test.

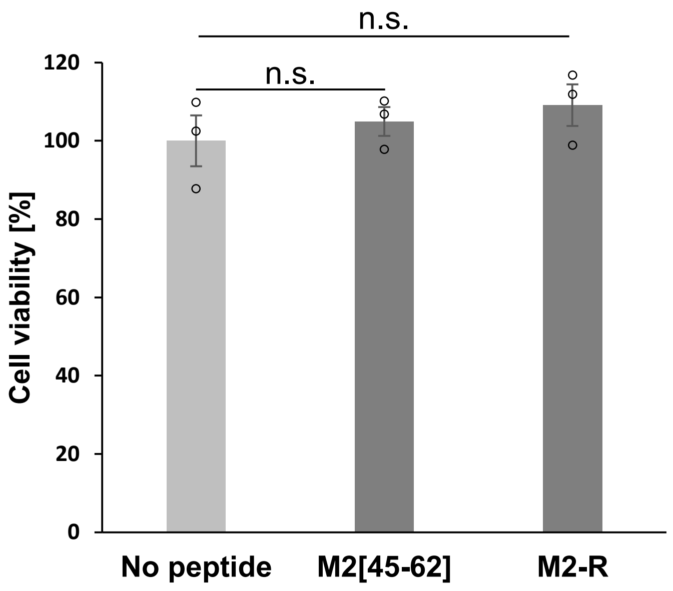

Supplementary Figure 12. No substantial cytotoxicity was observed in the M2[45–62] or M2-R treatments (WST-8 assay). The cells were starved in DMEM(-) for 6 h. COS-1 cells were then treated with M2[45–62] and M2-R (10 µM). The cells were incubated for another 18 h and then subjected to the WST-8 assay. Results are presented as means ± standard error (n = 3).

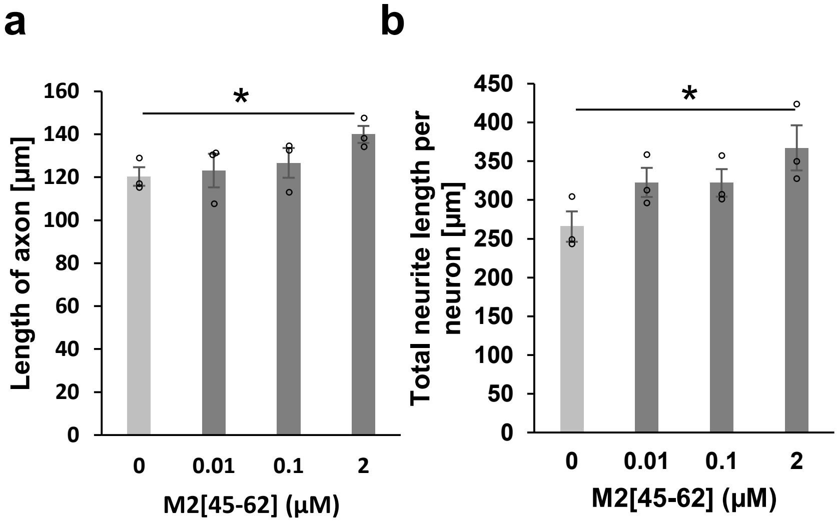

Supplementary Figure 13. M2[45–62] increases neurite outgrowth in cultured neurons. Quantification of the average length of the axons **(a)** and total neurite length per neuron (**b**) of the control and M2[45–62]-treated neurons. The mean ± standard error were derived from ~150 neurons pooled from three independent experiments. *$P < 0.05$, Student's t-test.

Supplementary Table 1. Hydrophobicity and net charges of the amphipathic peptides

	**Hydrophobicity <H>[a)]**	**Net charge**
**Bin1[8-33]**	**0.158**	**5**
**M2[45-62]**	**0.390**	**7**
**Epsin[1-18]**	**0.219**	**3**
**MIM[1-24]**	**0.488**	**-2**
**Arf[1-17]**	**0.429**	**2**
**dAmph[3-28]**	**0.127**	**4**
**SH3YL1[9-23]**	**0.109**	**3**

a) Mean hydrophobicity <H> was calculated by HeliQuest (http://heliquest.ipmc.cnrs.fr/) based on the procedures reported by Fauchère, J. & Pliska, V. *Eur J Med Chem*. 8, 369–375 (1983).

Supplementary Table 2. Reverse-phase high-performance liquid chromatography (HPLC) and matrix-assisted laser desorption ionization time-of-flight mass spectrometry (MALDI-TOF MS) analyses of each peptide.

Peptide	R_t (min)[a)]	m/z (obtained)	MS (calcd)[b)]
Bin1[8-33]	14.51	2808.057	2807.676
M2[45-62]	13.99	2350.675	2350.341
Epsin[1-18]	13.54	2115.495	2115.160
MIM[1-24]	23.83	2576.789	2576.382
Arf[1-17]	19.84	1895.150	1895.072
dAmph[3-28]	12.91	2885.698	2885.698
SH3YL1[9-23]	12.28	1731.337	1731.046
D-M2	14.26	2350.455	2350.341
Scr-M2	13.24	2350.587	2350.341
M2-R	14.48	2433.944	2434.360
M2-K	13.62	2237.901	2238.317
NBD-Bin1	17.08	2970.351	2970.678
NBD-M2	17.46	2513.077	2513.343
NBD-Epsin	17.63	2278.066	2278.162
NBD-MIM	28.76	2738.436	2739.384
NBD-Arf	24.16	2057.994	2058.074
NBD-dAmph	14.60	3048.668	3048.700
NBD-SH3YL1	16.35	1894.007	1894.048

a) Retention time (R_t) in HPLC (column: Cosmosil $_5C_4$-AR-300 (4.6 × 150 mm); gradient: 10–90% B in A (A = H_2O containing 0.1% CF_3COOH, B = CH_3CN containing 0.1% CF_3COOH) over 40 min; flow: 1 mL/min; detection: 220 nm); b) Calculated monoisotopic mass for $(M+H)^+$.
