# Supplementary material for: An influenza-derived membrane tension-modulating peptide regulates cell movement and morphology via actin remodeling
Source: Commun Biol. 2019 Jun 26;2:243. doi: 10.1038/s42003-019-0486-3 (PMC6594980; doi:10.1038/s42003-019-0486-3)
Supplement: Supplementary file 6 — Description of Additional Supplementary Files [file 42003_2019_486_MOESM6_ESM.docx]

Descriptions of additional supplementary files:

Supplementary Movie 1. Fluorescent speckle video of mRFP-actin in a growth cone in cultured neurons before adding M2[45–62]. The images were recorded every 5 seconds.

Supplementary Movie 2. Fluorescent speckle video of mRFP-actin in a growth cone in cultured neurons 30 min after adding M2[45–62]. The images were recorded every 5 seconds.
